# Closed-Loop Cardiovascular Interactions and the Baroreflex Cardiac Arm: Modulations Over the 24 h and the Effect of Hypertension

**DOI:** 10.3389/fphys.2019.00477

**Published:** 2019-05-07

**Authors:** Gianfranco Parati, Paolo Castiglioni, Andrea Faini, Marco Di Rienzo, Giuseppe Mancia, Riccardo Barbieri, J. Philip Saul

**Affiliations:** ^1^Department of Medicine and Surgery, University of Milano-Bicocca, Milan, Italy; ^2^Istituto Auxologico Italiano, IRCCS, Department of Cardiovascular, Neural and Metabolic Sciences, S.Luca Hospital, Milan, Italy; ^3^IRCCS Fondazione Don Carlo Gnocchi, Milan, Italy; ^4^Politecnico di Milano, Milan, Italy; ^5^West Virginia University School of Medicine, Morgantown, WV, United States

**Keywords:** ambulatory blood pressure monitoring, autonomic nervous system, arterial baroreflex, hypertension, blood pressure spectral analysis

## Abstract

Closed-loop models of the interactions between blood pressure (BP) and heart rate variations allow for estimation of baroreflex sensitivity (feedback effects of BP changes on heart rate) while also considering the feedforward effects of heart rate on BP. Our study is aimed at comparing modulations of feedback and feedforward couplings over 24 h in normotensive and hypertensive subjects, by assessing closed-loop baroreflex models in ambulatory conditions. Continuous intra-arterial BP recordings were performed for 24 h in eight normotensive and eight hypertensive subjects. Systolic BP (SBP) and pulse interval (PI) beat-by-beat series were analyzed by an autoregressive moving average model over consecutive 6-min running windows, estimating closed-loop feedback and feedforward gains in each window. The open-loop feedback gain was estimated for comparison. Normotensive and hypertensive patients were compared during wake (18:00–22:00) and sleep (23:00–5:00) periods by a mixed-effect linear model at *p* < 0.05. In both groups feedback (feedforward) gain averaged values were higher (lower) in sleep than in wake. Moreover, the closed-loop feedback gain was higher in normotensive subjects both in wake and sleep, whereas the closed-loop feedforward gain was higher in hypertensive subjects during sleep. By contrast, no significant differences were found between the normotensive and hypertensive groups for the open-loop feedback gain. Therefore, the closed-loop SBP-PI model can detect circadian alterations in the feedforward gain of PI on SBP and derangements of spontaneous baroreflex sensitivity in hypertension not detectable with the open-loop approach. These findings may help to obtain a more comprehensive assessment of the autonomic dysfunction underlying hypertension and for the in-depth evaluation of the benefits of rehabilitation procedures on autonomic cardiovascular modulation.

## Introduction

Animal and human studies have concordantly documented that the arterial baroreflex represents a fundamental mechanism to avoid excessive blood pressure (BP) oscillations and maintain its values within a range that preserves organ perfusion and avoids the risk associated with BP peaks (Mancia and Mark, 2011). Furthermore, data are available that in the clinical setting a baroreflex dysfunction may unveil the early occurrence of autonomic impairment in conditions such as arterial hypertension, diabetes, sleep apnea syndrome and aging, and predicts the risk of cardiovascular events in diseases like myocardial infarction, congestive heart failure or recurrent malignant arrhythmias (Parati et al., 2007, 2013).

Arterial baroreflex function can be assessed by delivering an external stimulus to baroreceptors and measuring the baroreflex-mediated response. Examples are the “Oxford” method, based on the intravenous injection of vasoactive drugs that induce reflex heart rate changes in response to drug-induced increases or reductions of systolic BP (SBP) (Smyth et al., 1969), and the “neck-chamber” method that stimulates or deactivates the carotid baroreceptors by respectively increasing or reducing carotid transmural pressure through changes in air pressure within a tight collar (Eckberg et al., 1975; Mancia et al., 1979). Other approaches are based on the analysis of baroreflex modulation of heart rate in response to the spontaneous fluctuations in BP which physiologically occur in daily life. At variance from the “Oxford” and “neck chamber” methods, such approaches avoid the inconveniences of the information obtained by delivery of external stimuli in the context of artificial laboratory settings and allow monitoring the baroreflex function in ambulant subjects for long periods (Mancia et al., 1983), without significantly interfering with their activities (Laude et al., 2004; Parati et al., 2004).

Both the laboratory and the “spontaneous” methods to study the arterial baroreflex estimate the feedback effects of SBP changes on pulse interval (PI), reciprocal of heart rate, neglecting the simultaneously occurring feedforward effects of PI on SBP, induced through changes in cardiac output. This can be acceptable under the “open-loop” assumption that these feedforward effects do not significantly influence the estimation of the gain of the feedback arc from SBP to PI. If the feedforward effects are not considered to be negligible, the open-loop assumption cannot be made and the feedforward effects of PI on SBP should be quantified simultaneously with the reflex feedback effects of SBP on PI. To date, “spontaneous” methods for evaluating the baroreflex function assessed feedback and feedforward effects simultaneously by mathematically modeling the beat-by-beat interactions among the cardiovascular variables through a closed-loop analysis of the time series (Barbieri et al., 1997, 2001). Simplified closed-loop models were based on bivariate autoregressive representations of the interactions between couples of cardiovascular time series (Barbieri et al., 1996), or on trivariate autoregressive models that also include respiratory signals (Baselli et al., 1988). Closed-loop auto-regressive moving average (ARMA) models of the SBP and PI beat-by-beat interactions were also proposed (Patton et al., 1996; Wyller et al., 2011). Applications of these models, however, have been limited to the laboratory environment only.

This work is focused on two primary aims. First, to characterize both the feedback and feedforward components of the SBP-PI coupling over 24 h in ambulatory subjects, which includes spontaneous variations of activity level during the day and night. Second, to compare the results between normotensive and hypertensive subjects to detect differences in their autonomic and vascular characteristics.

## Materials and Methods

### Subjects

The study utilized 24-h ambulatory intra-arterial BP recordings performed at the University Hospital (Ospedale Maggiore Policlinico di Milano) of Milan, Italy. Invasive recordings were used to obtain an uninterrupted BP signal over the 24 h, an advantage not offered by discontinuous ambulatory BP recorders which have also lower accuracy. Invasive monitoring provides more accurate BP data also when compared to non-invasive continuous BP recordings from devices based on the volume clamp method at the finger artery level (Castiglioni et al., 1999), which require periodic interruptions for calibration and for switching the measuring cuff between two fingers (Imholz et al., 1993). Intra-arterial BP recordings were obtained in eight normotensive subjects (five males and three females of which one in the childbearing age) and eight subjects with moderate to severe essential hypertension (seven males and one female in the childbearing age). Normotensive subjects were referred to our hospital for a suspected hypertensive state which was excluded by the clinical evaluation. Exclusion criteria were: (1) clinical or laboratory evidence of cardiovascular disease in addition to hypertension, (2) other significant health abnormalities (e.g., diabetes), (3) smoking, (4) obesity, (5) prior drug treatment for hypertension, and (6) administration of cardiovascular drugs in the 4 weeks preceding the BP recording. To be included for analysis, the BP signal had to be of sufficiently high quality over the entire 24-h period.

Subjects were classified as normotensive or hypertensive by averaging three systolic and diastolic (D) BP values collected at 5 min intervals in the sitting position, after a 5 min rest, using a mercury sphygmomanometer in each of two visits, scheduled at 1-month intervals. The normotensive subjects had mean (SD) SBP and DBP values of 131 (6) and 84 (4) mmHg respectively, while the hypertensive subjects had corresponding values of 191 (19) and 104 (7) mmHg. Ages of the two groups were statistically similar: 43 (20) years (range: 19–70) for normotensive subjects vs. 50 (15) years (range: 28–67) for hypertensive subjects. No subject had any alteration in glucose metabolism or renal function. The study was carried out following the recommendations of the Ospedale Maggiore Policlinico di Milano (Milan, Italy) ethical committee with written informed consent from all subjects in accordance with the Declaration of Helsinki. The protocol was approved by the Ospedale Maggiore Policlinico di Milano (Milan, Italy).

### Measurements Protocol

A catheter (11 cm long, 1.1 mm internal diameter) was percutaneously inserted into the radial artery of the non-dominant arm by the Seldinger technique after local anesthesia with 2% lidocaine. A rigid polyethylene tube connected the catheter to a transducing-perfusing unit contained in a plexiglass box secured to the patient’s thorax at the heart level. The BP signal was stored on a magnetic tape cassette by an Oxford Medilog recorder bound to the subjects’ waist. The method provides an accurate BP recording because of the stability of the zero signal, the transducer linearity between 50 and 250 mmHg, and the undistorted frequency-response up to 10 Hz (Stott et al., 1976).

Ambulatory recordings started around 6 pm and ended at 6–7 pm on the following day. Meal times, bed times and recreational times (T.V. watching, playing cards, visits from relatives) were standardized. Meals composition was also standardized and provided by the hospital canteen. Subjects were allowed to move within the hospital buildings and garden, but not outside the hospital area. They were asked to record their activities in a diary and were discouraged from performing any kind of vigorous physical exercise.

### Data Analysis

The recorded BP signals were digitized (170 Hz, 12 bits), manually edited from movement artifacts, pulse pressure dampening, and premature beats. SBP was calculated for each pulse wave beat-by-beat and PI of a given beat “n” was computed as the interval between the times of occurrence of the systolic peak of the beat “n” and of the systolic peak of the successive beat, “n+1,” as described previously (Di Rienzo et al., 2006). For the closed-loop analysis, the beat series were re-sampled at 3 Hz, high-pass filtered (corner frequency of 0.03 Hz) to remove very-low frequency components, and split into contiguous segments of 1024 samples (about 6 min). The SBP and PI variances in each 6-min segment were calculated and averaged over the entire recording as measures of SBP and PI short-term variability.

The feedback and feedforward components of the SBP-PI coupling were estimated using the following bivariate closed-loop autoregressive model in each segment:

(1) $$\left[\begin{array}{c} PI(n)\\ SBP(n) \end{array}\right]=\sum_{k=1}^{p}\left[\begin{array}{cc} a_{11}(k) & a_{12}(k)\\ a_{21}(k) & a_{22}(k) \end{array}\right]\;\times \left[\begin{array}{c} PI(n-k)\\ SBP(n-k) \end{array}\right]+\left[\begin{array}{l} w_{PI}(n)\\ w_{SBP}(n) \end{array}\right]$$

with 1 ≤*n* ≤ 1024, *w_PI_* and *w_SBP_* representing independent white Gaussian noises, and the model order *p* set equal to 14 to guarantee a model order higher than the minimum required by the Akaike criterion. The *a_ji_(k)* coefficients were estimated by the Levinson-Wiggins-Robinson algorithm (Wiggins and Robinson, 1967).

The feedback transfer function between SBP and PI, *G_SBP→PI_*, and the feedforward transfer function between PI and SBP, *G_PI→SBP_*, were estimated as:

(2) $$G_{SBP\to PI}(f)=\frac{A_{12}(f)}{1-A_{11}(f)}$$

(3) $$G_{PI\to SBP}(f)=\frac{A_{21}(f)}{1-A_{22}(f)}$$

(4) $$\text{with }A_{ij}(f)=\sum_{k=1}^{p}a_{ij}(k)e^{-j2\pi fk}$$

The absolute values of feedback and feedforward transfer functions were computed in the low frequency (0.04–0.15 Hz, LF) and high frequency (0.15–0.5 Hz, HF) bands. The absolute value of closed-loop feedback gain, hereafter α_C_, was taken as the measure of closed-loop baroreflex sensitivity on PI. The absolute value of the closed-loop feedforward gain, hereafter β_C_, was taken as a measure of the sensitivity of the mechanical coupling between PI and SBP. Estimates of SBP-PI coupling were considered reliable only for data segments with SBP-PI squared coherence modulus greater than 0.5, which occurred almost exclusively in the LF band, leading to the exclusive use of the LF band for the SBP-PI relationships.

Since traditional methods for estimating the cardiac arm of the baroreflex with transfer function techniques do not consider the closed-loop nature of the baroreflex, the ratio between SBP-PI cross-spectrum and SBP spectrum

(5) $$H_{SBP\to PI}(f)=\frac{P_{SBP-PI}(f)}{P_{SBP}(f)}$$

was also calculated over each 1024-point data segment, to evaluate how neglecting the closed-loop nature of the cardiac baroreflex influences the estimation of the feedback component. The open-loop feedback gain, α_O_, was then estimated as the modulus of *H_SBP→PI_* transfer function in the LF band. Values estimated over each 6-min running window (without overlapping) were averaged hour by hour to obtain hourly profiles over the 24 h. Moreover, spectral indices associated with autonomic cardiovascular control were computed. The LF power of SBP, both in absolute units and in normalized units, [LF/(LF+HF)], was calculated as an index of vasomotor sympathetic tone. The PI power in the HF band and the ratio between LF and HF powers of PI, LF/HF powers ratio, were calculated as indices of cardiac vagal modulation and of cardiac sympatho/vagal balance.

### Statistical Analysis

Based on previous evidence (Castiglioni et al., 1999), spectral powers were log-transformed to reduce the skewness of their distribution. Gaussianity of log-transformed spectral powers and of feedback and feedforward baroreflex gains was verified by the Shapiro–Wilk’s normality test. 24-h estimates were compared between normotensive and hypertensive subjects by the two-sided *t*-test. The two groups were also compared over shorter time periods selected as being more likely associated with higher and lower degrees of sympathetic activation, respectively. The segment between 6 and 10 pm was selected as the sub-period with a higher sympathetic activation because it followed the stress related to the subject’s invasive instrumentation. Subjects were awake and not lying in bed by their diaries, so this period was labeled “wake.” The segment between 11 pm and 5 am was selected as the sub-period with lower sympathetic activation. Subjects were asleep according to their diaries, so this period is labeled “sleep.” Normotensive and hypertensive groups were compared over the above “wake” and “sleep” subperiods considering one “between” factor (group factor) and one “within” factor (time factor). Significances of each of the two factors and of their interaction (time × group) were calculated by applying a mixed-effect linear model, with *post hoc* contrast analysis corrected for multiple comparisons by the Benjamini and Hochberg False Discovery Rate procedure.

Circadian/ultradian modulations were statistically described by hourly profiles of log-transformed feedback and feedforward gains. The relationship of either feedback and feedforward gains with BP and PI short-term variability was described by linear regression analysis with α_C_ or β_C_ calculated over the whole 24-h period as independent variables, and the average over the 24-h period of SBP or PI short-term variances (as assessed over the running window of 6 min employed for ARMA analysis) as dependent variables.

Analyses were performed with R Statistical package (The R Foundation for Statistical Computing, Vienna, Austria) setting the significance threshold at *p* < 0.05. The beat-by-beat series recorded in this study are available from the corresponding author on reasonable request.

## Results

Table 1 shows that compared to normotensive subjects, hypertensive subjects exhibited, over the 24 h, higher BP values, as well as a higher SBP variance. Furthermore, compared to normotensive subjects, hypertensive subjects had a lower baroreflex feedback gain (the difference, however, reaching the statistical significance when calculated by the closed-loop approach, α_C_, and not by the open-loop approach, α_O_) and a tendency (*p* = 0.06) toward a higher feedforward gain, β_C_.

**Table 1:** 24-h BP and heart rate mean, variance and spectral indices and 24-h feedback/feedforward baroreflex gains: mean (SD) with p significance of the difference between normotensive and hypertensive groups.

	Normotensive	Hypertensive	*p*-value
**Mean**			
SBP (mmHg)	120.8 (18.9)	170.9 (20.1)	**<0.001**
DBP (mmHg)	65.4 (11.2)	87.7 (16.2)	**<0.01**
Heart rate (bpm)	77.6 (5.4)	77.2 (12.8)	0.93
**SBP variability**			
Variance (mmHg^2^)	229.4 (82.4)	395.6 (121.1)	**<0.01**
LF power (mmHg^2^)	15.61 (6.69)	20.54 (8.85)	0.20
LF normalized power	0.50 (0.09)	0.45 (0.11)	0.38
**PI variability**			
Variance (ms^2^)	19015 (10780)	13934 (7827)	0.38
HF power (ms^2^)	426.9 (448.6)	171.6 (120.5)	0.28
LF/HF powers ratio	2.2 (1.7)	3.0 (2.3)	0.45
**Baroreflex gains**			
α_O_ (ms/mmHg)	7.19 (3.75)	4.13 (1.29)	0.0504
α_C_ (ms/mmHg)	3.33 (1.90)	1.75 (0.63)	**<0.05**
β_C_ (mmHg/ms)	0.12 (0.02)	0.17 (0.02)	0.06

*p after two-sided *t*-test (the bold font highlights significances at *p* < 0.05); SBP, systolic blood pressure; DBP, diastolic blood pressure; LF, low frequency; HF, high frequency; α_*O*_, feedback gain (open-loop model); α_*C*_, feedback gain (closed-loop model); β_*C*_, feedforward gain (closed-loop model). Variance, LF power, HF power, LF/HF powers ratio, and feedback/feedforward gains were log-transformed before the statistical test.*

### Closed-Loop Gains vs. Short-Term Variability

**FIGURE 1:**
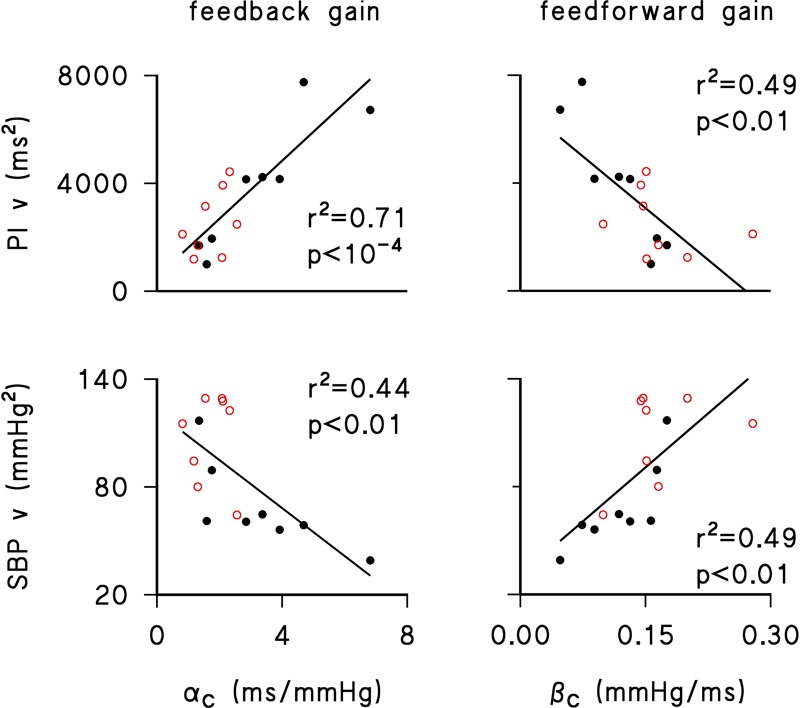
Linear regression between feedback gain (α_C_, *left panels*) or feedforward gain (β_C_, *right panels*) and short-term variance of PI (PI v, *upper panels*) or SBP (SBP v, *lower panels*) for normotensive (black closed circles) and hypertensive (red open circles) individuals. Determination coefficients, r^2^, and significance of linear trends, p, are reported in each panel. Short-term variances were calculated by averaging, over 24 h, the variance of SBP or PI values in each 6-min running window employed for assessing α_C_ and β_C_.

The regression analysis (Figure 1) shows that both feedback and feedforward gains were linearly related to short-term variability of both PI or SBP (i.e., the variance over a running window of 6 min). In particular, higher feedback gains were linearly associated with higher PI variances and lower SBP variances, while higher feedforward gains were linearly associated with lower PI variances and higher SBP variances. These observations were similar in the normotensive and hypertensive groups (Figure 1).

### Wake vs. Sleep Subperiods

Table 2 shows that indices of vascular sympathetic modulation (LF power of SBP) and of cardiac sympatho/vagal balance (LF/HF powers ratio of PI) were higher in the *wake* than in the *sleep* subperiods, while the reverse was true for the index of cardiac vagal modulation (HF power of PI). Differences between wake and sleep subperiods in the PI spectral indices were more pronounced in the normotensive group, even if the “group” factor and the interaction between factors did not reach the statistical significance.

**Table 2:** Autonomic spectral indices in “wake” and “sleep” periods: mean (SD) and significance p of the factors time and group (abbreviations as in Table 1).

	Wake	Sleep	*p*-value		
			Time	Group	Time × group
**SBP LF (mmHg^2^)**					
Normotensive	21.2 (8.1)^∗^	8.5 (6.1)	**<0.001**	0.434	0.991
Hypertensive	30.5 (21.9)^∗^	9.2 (3.5)			
**SBP normalized LF**					
Normotensive	0.54 (0.08)^∗^	0.39 (0.11)	**<0.001**	0.136	0.454
Hypertensive	0.48 (0.12)^∗^	0.30 (0.10)			
**PI HF (ms^2^)**					
Normotensive	246 (240)^∗^	768 (904)	**0.001**	0.255	0.247
Hypertensive	118 (67)°	235 (166)			
**PI LF/HF**					
Normotensive	2.7 (0.6)^∗^	1.6 (1.1)	**0.002**	0.411	0.286
Hypertensive	3.7 (3.1)°	2.1 (1.0)			

*Factors significance by mixed model analysis (the bold font highlights significances at *p* < 0.05): the “^∗^” and “°” symbols mark differences between “wake” and “sleep” periods significant at *p* < 5% and *p* < 10% after contrast analysis. LF powers, HF powers and the LF/HF powers ratio were log-transformed before statistical analysis.*

**FIGURE 2:**
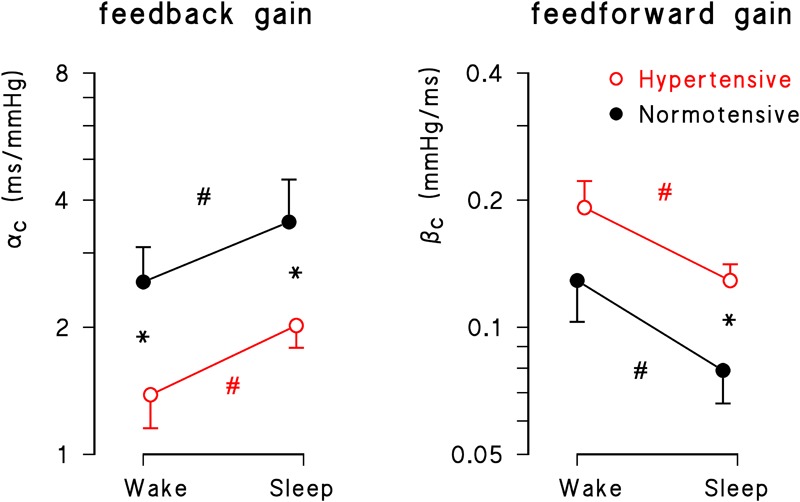
Geometric mean ± geometric standard error for feedback (α_C_) and feedforward (β_C_) gains during *wake* and *sleep* periods in normotensive (solid black circle) and hypertensive (open red circle) groups. Asterisks indicate significant differences between groups; number signs, # indicate significant differences between conditions from the mixed-effect linear model analysis.

*Wake* and *sleep* sub-periods differed markedly also for closed-loop feedback and feedforward gains (Figure 2). The feedback gain was significantly higher and the feedforward gain was significantly lower during *sleep* (*p* = 10^-3^ for factor “time” for both gains). The “group” factor reached statistical significance for both feedback (*p* = 0.03) and feedforward (*p* = 0.04) gains, and the hypertensive group had significantly lower feedback gains in *wake* and *sleep* conditions and significantly higher feedforward gains during *sleep*.

Also for the open-loop estimates of the feedback gain the factor “time” was significant (*p* = 10^-4^), being α_O_ greater in *sleep* than in *wake* conditions both for normotensive (wake: 5.21 ± 3.25; sleep: 9.38 ± 5.44 ms/mmHg, *p* < 0.05) and hypertensive participants (wake: 3.11 ± 1.29; sleep: 5.26 ± 1.69 ms/mmHg, *p* < 0.05). However, differently from the results obtained for α_C_, the factor “hypertension” fell short of statistical significance (*p* = 0.06).

### 24-h Profiles

**FIGURE 3:**
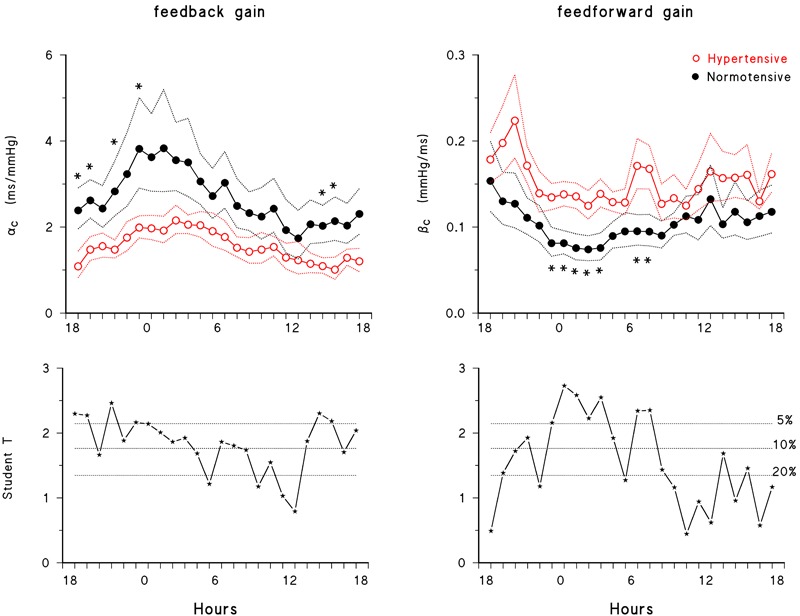
24-h closed-loop profiles. **(Upper)** Feedback and feedforward gains in normotensive (solid black circle) and hypertensive (open red circle) subjects: geometric mean ± geometric standard error, with ^∗^ indicating significant differences (*p* < 0.05) between normotensive and hypertensive groups (unpaired *t*-test). **(Lower)** Student’s *t*-test statistics for the difference between groups, with dotted horizontal lines representing the thresholds at 20, 10, and 5% significance.

Figure 3 describes the circadian/ultradian modulations of closed-loop gains in normotensive subjects and their alterations with hypertension. The feedback gain showed a clear night/day modulation with greater gains at night time. The 24-h closed-loop gain profile was higher in normotensive than in hypertensive subjects and the statistical significance between groups was achieved between early afternoon and midnight, progressively decreasing from midnight to noon.

The feedforward gain had a different profile. That is, it also showed a night/day modulation, but with lower values at night. Furthermore, it was greater in hypertensive subjects, with between-group differences that achieved the maximal statistical significance mainly at night. After 7 am, differences between groups decreased and almost vanished between 12 and 6 pm, in line with the mixed-effect linear model analysis that found a significant difference between hypertensive and normotensive subjects in *sleep* only (Figure 2). Significant differences between groups were also found around the awakening period. While the feedforward gain rose smoothly from 3 to 5 am in normotensive subjects, in hypertensive subjects it showed a rather constant “plateau” between 11 pm and 5 am, followed by a peak around the wake-up time, between 6 and 7 am.

**FIGURE 4:**
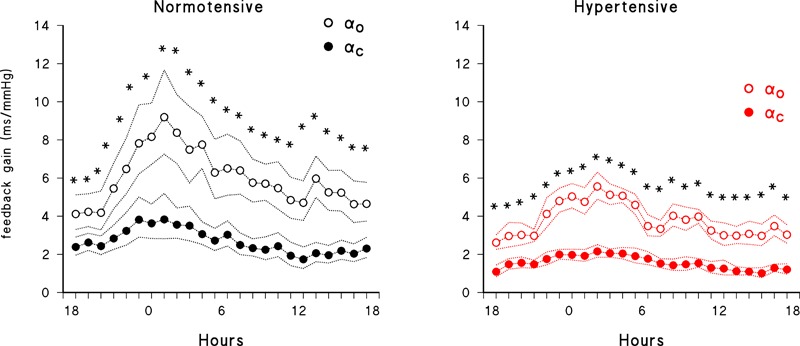
Comparison of 24-h closed-loop (solid circles) and open-loop (open circles) feedback gains. Geometric mean ± geometric standard error in normotensive (black) and hypertensive (red) subjects; asterisks indicate significant (*p* < 0.05) differences between open- and closed-loop gains by paired *t*-test after log-transformation.

Figure 4 compares the 24-h profiles of open- and closed-loop estimates. It shows similar circadian modulations with, however, closed-loop estimates always consistently lower than open-loop estimates, in both groups of participants.

## Discussion

This study provides the first closed-loop quantitative characterization of the coupling between PI and SBP over the 24 h in ambulant subjects, separately for the feedback reflex component (heart rate modulations in response to BP changes) and for the feedforward mechanical component (BP variations in response to heart rate changes). It also compares, for the first time, closed-loop PI-SBP gains between normotensive and hypertensive subjects throughout the 24 h. Finally, it quantifies differences between closed- and open-loop estimates of baroreflex gain both at normal and high BPs. The following specific results deserve to be discussed.

### Correlation of Feedback Gain With SBP and PI Variability

First, the sensitivity of the baroreflex-PI reflex, as quantified by the closed-loop feedback gain over the 24 h, correlated positively with PI variability and negatively with SBP variability. This is in line with similar correlations observed by assessing baroreflex sensitivity through injections of phenylephrine or nitroglycerin (Mancia et al., 1986).

### Circadian Modulation of the Feedback Gain and Hypertension

Second, our participants exhibited a higher value of the closed-loop feedback gain during sleep than in the awake state, and the feedback gain was lower in hypertensive than in normotensive subjects over the entire 24-h period, with a difference that was more consistent during the day than during the night time. These findings extend to a closed-loop analysis of real-life observations previous reports on *wake*/*sleep* modulation and on the difference between normotensive and hypertensive subjects obtained in the laboratory from the PI responses to phenylephrine injections (Conway et al., 1983) and by a time-domain open-loop method, the “sequence technique” (Parati et al., 1988). Of note is the present observation that the awake-sleep modulation of the baroreflex gain, although reduced, is not suppressed in hypertension. Ultradian modulation of the baroreflex and its lower sensitivity during the day time have been attributed to central neural influences on the baroreflex arch (Di Rienzo et al., 2009). Changes of the baroreflex gain associated with the lying body position during sleep may also be involved, however, because higher baroreflex sensitivity in supine than in standing posture has been reported with different estimation methods (Laude et al., 2004).

### Circadian Modulation of the Feedforward Gain

Third, this study provides novel information on the 24-h modulation of the feedforward gain. The mechanical coupling from PI to SBP had higher gain during *wake* than during *sleep* (Figure 2), probably because of a higher cardiac and vascular sympathetic activity in the awake period than at night, as suggested by previous studies (Di Rienzo et al., 1989; Furlan et al., 1990; Parati et al., 1990) but also by the present finding of greater LF power of SBP and LF/HF powers ratio of PI during the day time. A higher sympathetic activity may amplify, via changes in myocardial contractility, the effect of heart rate changes on cardiac output and vascular distensibility (Giannattasio et al., 2005), increasing the mechanical coupling between heart rate and BP. Interestingly, while the feedback gain correlated negatively with SBP variability (reflecting the baroreflex “buffering” action on BP fluctuations) the feedforward gain and SBP variability showed a positive relationship. This is consistent with the possibility that, as the feedforward gain increases, the same PI variations produce larger SBP variations.

### Hypertension and Feedforward Gain

Fourth, our study provides novel information on alterations of the feedforward gain in hypertension. The gain of the PI-SBP mechanical coupling tended to be higher in hypertensive subjects. Higher gain is consistent with the vascular alterations characterizing hypertension, such as decreased arterial distensibility and increased systemic vascular resistance (Mitchell et al., 2008), the presence of which may amplify the effect of changes in cardiac output (majorly dependent on heart rate) on BP. The differences between hypertensive and normotensive subjects were particularly significant during “sleep” (Figure 2, 3), which might indicate an impaired capability of the hypertensive group to deactivate the mechanical PI-SBP coupling at night, an impairment which seems related to their known structural vascular changes responsible for an increased arterial stiffness, and to their lower capability to enhance vagal control and to reduce the cardiac sympatho/vagal balance at night (Table 2). It is worth noting that the profiles of the feedforward gains of normotensive and hypertensive subjects differed markedly in the hours immediately after awakening, between 6 and 8 am. The β_C_ peak visible in the hypertensive group only in this time window (Figure 3) suggests a faster rise of the feedforward gain in hypertensive subjects after wake-up. This finding might be related with the so-called morning BP surge and with the associated greater incidence of cardiovascular events after awakening reported in previous studies (Muller et al., 1989; Kario et al., 2003), an issue which deserves to be further investigated in future studies.

### Open vs. Closed Loop Estimates

A last methodological point regards the influence of feedforward components when estimating the feedback gain. Feedback gains are substantially lower if estimated by closed-loop rather than by open-loop models. Open-loop techniques might disregard the influences of PI changes on changes in SBP and therefore ascribe all PI fluctuations in the frame of SBP-PI coupling to reflex influences triggered by changes in BP. For instance, the open loop approach does not take into account that, when PI lengthens due to the reflex effects of an elevation in SBP, it consequently leads to a reduction in SBP. Since also secondary changes in SBP may be linearly coupled to changes in PI, the mechanical gain from PI to SBP would reasonably lead to a bias toward higher feedback gains from SBP to PI. In our study, in spite of the relatively small number of subjects included, the more precise evaluation allowed by the closed-loop approach detected significant differences in baroreflex sensitivity between the normotensive and hypertensive groups which is in line with previous data obtained with vasoactive drugs (Conway et al., 1983). Conversely, the open-loop method, although showing a clear tendency toward a between groups difference, failed to cross the significance threshold.

### Limitations

The use of invasive BP recordings more faithfully describes the beat-by-beat cardiovascular dynamics than non-invasive methods. Therefore, the methodology in this study needs to be adapted and validated in other settings where non-invasive recordings are considered. In fact, the use of an intra-arterial catheter for BP measurements is generally limited now to BP recordings in intensive care units, and non-invasive methods measuring BP at the digital artery level are preferred for monitoring free-moving subjects over the 24 h. Due to the peripheral measurement sites, the LF powers of SBP are amplified when measured at the finger artery level (Omboni et al., 1993; Castiglioni et al., 1999). Therefore, closed-loop estimates of the gains in the cardiac baroreflex loop obtained with these non-invasive methods might differ from those reported in the present work. A technical limitation to be mentioned is that we did not simultaneously record the electrocardiogram or any respiratory signal. Since the electrocardiogram was not measured, the baroreflex modulation of heart rate was quantified from PI measures, while R–R intervals are expected to more faithfully reflect the autonomic modulations of heart rate. However, discrepancies between PI and R–R intervals variability are negligible in comparison to the linear relationship between the two variables and BP, and mostly present in the frequency range occupied by the respiratory fluctuations only (Constant et al., 1999). We may therefore reasonably assume that our results, based on the slower oscillations in the LF band, are not substantially influenced by the choice between PI or R–R intervals. The lack of a respiratory signal means that our bivariate model cannot quantify how respiration influences the SBP and PI series. With a respiratory signal available, a trivariate ARMA model is likely to estimate the feedback and feedforward SBP-PI relations more precisely. However, a comparison of closed-loop baroreflex gains by a bivariate AR model of SBP and R–R intervals and by a trivariate AR model that also included a respiratory signal provided substantially similar results (Barbieri et al., 1997).

As a final methodological comment, it should be mentioned that, even if quantifying separately the feedforward and feedback components of the SBP-PI interactions, our closed-loop analysis does not measure the exact directional importance of the causality relation between the two series. Future closed-loop models including the concept of Granger causality within the assessment of the closed-loop relations might provide further details into the estimation of the feedforward and feedback transfer functions.

## Conclusion

The removal of the open-loop assumption when modeling the interaction between BP and heart rate fluctuations offers a deeper insight into the mechanisms involved in daily life cardiovascular regulation. In particular, it allows the detection of specific patterns characterizing the altered cardiovascular regulation reported in essential hypertension separately for feedback and feedforward gains. Such closed-loop evaluation may improve the clinical relevance of SBP-PI coupling assessment over the 24 h, by separately quantifying the contribution of the baroreflex feedback gain and of the mechanical feedforward coupling between SBP and PI in relation to target organ damage, incidence of cardiovascular events and efficacy of treatments in hypertension, a possibility which deserves to be specifically explored in future studies.

## Ethics Statement

The study was carried out in accordance with the recommendations of the Ospedale Maggiore Policlinico di Milano (Milan, Italy) ethical committee with written informed consent from all subjects. All subjects gave written informed consent in accordance with the Declaration of Helsinki. The protocol was approved by the Ospedale Maggiore Policlinico di Milano (Milan, Italy).

## Author Contributions

GP and JS conceived the study. RB and PC performed the data analysis. AF performed the statistical analysis. GP and PC wrote the manuscript. All the authors fulfilled data interpretation and critically revised the manuscript.

## Conflict of Interest Statement

The authors declare that the research was conducted in the absence of any commercial or financial relationships that could be construed as a potential conflict of interest.
